# Supplementary material for: Coordination Sites for Sodium and Potassium Ions in Nucleophilic Adeninate Contact ion-Pairs: A Molecular-Wide and Electron Density-Based (MOWED) Perspective
Source: Molecules. 2022 Sep 19;27(18):6111. doi: 10.3390/molecules27186111 (PMC9505275; doi:10.3390/molecules27186111)
Supplement: Supplementary file 1 [file molecules-27-06111-s001.zip › molecules-1911464-supplementary.pdf]

Coordination Sites for Sodium and Potassium Ions in Nucleophilic Adeninate Contact ion-Pairs: A Molecular-Wide and Electron Density-Based (MOWED) Perspective

Dominique M.S. Buyens , Lynne A. Pilcher and Ignacy Cukrowski *

Department of Chemistry, University of Pretoria, Pretoria 0002, Republic of South Africa;
* Correspondence: ignacy.cukrowski@up.ac.za

Supplementary Materials

Table of contents: Page

Additional CCSD data for the implicit solvation model 2

Additional DFT data for the implicit solvation model 4

Additional Figures and Tables for the explicit solvation model 9

Additional CCSD data for the implicit solvent model

Table S1: CCSD-computed intermolecular diatomic interaction energies between **Na⁺** with the atoms of the adeninate anion for the specified Na-Ade complexes (CIPs). All values in kcal mol⁻¹

	CIPs						
**Atom**	**N3N9**	**N9**	**N3**	**N7**	**N1**	**N10**	**π**
N1	-78.3	-68.3	-82.8	-71.8	-177.9	-102.6	-93.1
C2	93.1	74.6	102.1	59.1	105.7	72.9	83.4
N3	-161.9	-114.6	-181.0	-67.3	-88.5	-73.5	-101.2
C4	111.3	95.9	100.6	59.9	55.8	55.9	76.6
C5	28.2	27.0	25.5	40.5	24.1	30.1	29.4
C6	58.3	52.9	58.5	71.7	98.8	94.5	75.0
N7	-79.8	-82.0	-71.7	-179.3	-64.3	-92.0	-103.0
C8	86.9	92.2	71.4	96.9	48.5	61.3	81.6
N9	-173.0	-182.7	-124.2	-84.7	-60.1	-66.9	-103.2
N10	-56.1	-52.8	-55.7	-92.7	-100.2	-155.0	-81.6
H11	18.2	16.9	18.3	27.6	39.3	43.7	24.7
H12	18.9	18.1	18.5	37.9	29.2	44.1	26.4
H13	3.1	2.1	3.2	1.7	2.5	2.2	2.3
H14	3.0	2.8	2.1	2.7	1.4	1.7	2.2
Total	***-128.0***	***-117.8***	***-115.1***	***-97.8***	***-85.6***	***-83.5***	***-80.5***

Table S2: CCSD-computed intermolecular diatomic interaction energies between **K^+** with the atoms of the adeninate anion for the specified K-Ade complexes (CIPs). All values in kcal mol^{-1}

	CIPs			
**Atom**	**N3N9**	**N7**	**N1**	**N10**
N1	-74.9	-68.1	-158.8	-158.8
C2	87.8	56.1	96.7	96.7
N3	-152.1	-64.3	-83.9	-83.9
C4	99.5	56.5	53.0	53.0
C5	26.1	36.2	22.6	22.6
C6	55.4	66.6	89.9	89.9
N7	-75.1	-160.2	-61.7	-61.7
C8	79.4	88.9	46.6	46.6
N9	-154.7	-80.6	-57.9	-57.9
N10	-53.8	-86.4	-93.9	-93.9
H11	17.5	25.9	35.9	35.9
H12	18.1	34.4	27.5	27.5
H13	2.7	1.6	1.9	1.9
H14	2.5	2.1	1.3	1.3
Total	***-121.6***	***-91.4***	***-80.7***	***-80.7***

Additional DFT/B3LYP data for the implicit solvation model

Table S3: DFT-computed intermolecular diatomic interaction energies between **Na$^+$** with the atoms of the adeninate anion for the specified Na-Ade complexes (CIPs). All values in kcal mol^{-1}

	CIPs						
**Atom**	**N3N9**	**N9**	**N3**	**N7**	**N1**	**N10**	**π**
N1	-73.4	-63.6	-77.0	-66.5	-142.1	-97.5	-97.5
C2	86.8	68.7	93.7	54.0	85.6	67.7	87.1
N3	-161.4	-110.0	-176.9	-62.7	-75.6	-69.3	-109.4
C4	104.4	90.0	93.1	55.5	47.6	52.1	75.4
C5	26.7	25.6	24.0	38.3	21.0	28.5	27.4
C6	54.1	48.7	53.8	66.2	82.6	87.2	73.2
N7	-74.2	-76.4	-66.7	-175.4	-57.7	-86.1	-93.3
C8	78.2	83.4	64.2	87.5	41.5	55.2	73.1
N9	-164.7	-178.2	-116.4	-78.7	-53.1	-62.5	-99.7
N10	-54.3	-50.9	-53.7	-89.5	-91.9	-150.9	-79.1
H11	18.4	17.0	18.4	27.7	38.2	43.8	27.1
H12	19.1	18.2	18.5	38.3	28.2	43.9	28.2
H13	3.3	2.2	3.3	1.7	2.2	2.3	2.8
H14	3.4	3.4	2.4	3.4	1.4	2.0	2.6
Total	***-133.6***	***-122.1***	***-119.4***	***-100.1***	***-72.1***	***-83.8***	***-82.3***

Table S4: DFT-computed intermolecular diatomic interaction energies between **K**$^{+}$ with the atoms of the adeninate anion for the specified K-Ade complexes (CIPs). All values in kcal mol^{-1}

	CIPs			
**Atom**	**N3N9**	**N7**	**N1**	**N10**
N1	-68.9	-63.9	-148.3	-103.7
C2	79.3	51.8	86.6	73.6
N3	-143.5	-60.4	-77.1	-76.4
C4	90.8	52.4	48.5	56.2
C5	24.3	34.2	21.2	27.3
C6	50.4	62.2	82.1	83.5
N7	-69.6	-152.8	-57.9	-85.9
C8	71.1	79.2	41.8	57.4
N9	-145.8	-74.9	-53.6	-67.2
N10	-51.3	-84.9	-91.0	-128.9
H11	17.4	26.2	36.2	38.0
H12	18.0	35.0	27.6	37.7
H13	2.7	1.6	2.1	2.5
H14	2.8	2.7	1.5	2.1
Total	***-122.2***	***-91.5***	***-80.2***	***-83.8***

Influence of the CIP formation on the intramolecular diatomic covalent (CB) and long-distance (LD) interactions between non-bonded atoms of the adeninate anion: DFT data

Table S5: DFT-computed changes in the total intramolecular interaction energy of the adeninate anion ($\Delta E_{\mathrm{int}}^{\mathrm{Ade}^-}$), the total CB-interactions ($\Delta^{\mathrm{CB}} E_{\mathrm{int}}^{\mathrm{Ade}^-}$) and total LD-interactions ($\Delta^{\mathrm{LD}} E_{\mathrm{int}}^{\mathrm{Ade}^-}$) calculated for the indicated Na-Ade and K-Ade complexes. All values in kcal mol^{-1}

	**CIPs**						
	**(N3N9)**	**(N9)**	**(N3)**	**(N7)**	**(N1)**	**(N10)**	**(π)**
	**Na-Ade complexes**						
$\Delta E_{\mathrm{int}}^{\mathrm{Ade}^-}$	–32.5	–22.2	–23.3	–12.3	–8.1	20.2	1.0
$\Delta^{\mathrm{CB}} E_{\mathrm{int}}^{\mathrm{Ade}^-}$	–43.1	–29.1	–30	–14.2	–9.3	31.6	1.8
$\Delta^{\mathrm{LD}} E_{\mathrm{int}}^{\mathrm{Ade}^-}$	10.6	7.0	6.7	1.9	1.2	–11.4	–0.8
	**K-Ade complexes**						
$\Delta E_{\mathrm{int}}^{\mathrm{Ade}^-}$	–19.1	-	-	–3.3	–0.7	14.9	-
$\Delta^{\mathrm{CB}} E_{\mathrm{int}}^{\mathrm{Ade}^-}$	–24.5	-	-	–2.5	2	24	-
$\Delta^{\mathrm{LD}} E_{\mathrm{int}}^{\mathrm{Ade}^-}$	5.4	-	-	–0.8	–2.7	–9.1	-

Table S6: The total change in the exchange correlation ($\Delta^{\mathrm{CB}} V_{\mathrm{XC}}^{\mathrm{Ade}^-}$) and classical ($\Delta^{\mathrm{CB}} V_{\mathrm{cl}}^{\mathrm{Ade}^-}$) terms of the interactions between covalently bonded atoms of the adeninate anion, calculated for Na-Ade and K-Ade complexes at the DFT level. All values in kcal mol^{-1}

	**CIPs**						
	**(N3N9)**	**(N9)**	**(N3)**	**(N7)**	**(N1)**	**(N10)**	**(π)**
	**Na-Ade complexes**						
$\Delta^{\mathrm{CB}} V_{\mathrm{XC}}^{\mathrm{Ade}^-}$	6.0	4.2	4.6	3.5	2	2.3	0.5
$\Delta^{\mathrm{CB}} V_{\mathrm{cl}}^{\mathrm{Ade}^-}$	–41.9	–33.3	–34.6	–17.8	–11.3	29.3	1.3
	**K-Ade complexes**						
$\Delta^{\mathrm{CB}} V_{\mathrm{XC}}^{\mathrm{Ade}^-}$	4.4	-	-	2.2	1.9	1.6	-
$\Delta^{\mathrm{CB}} V_{\mathrm{cl}}^{\mathrm{Ade}^-}$	–28.9	-	-	–4.7	0	22.3	-

Change in net atomic charges upon CIP formation – DFT data

Table S7: Net atomic charges of the atoms Q(A) of free Ade⁻, the net molecular charge of Ade⁻ Q(Ade⁻) and counter ions $Q(Na^+)$ and $Q(K^+)$. Relative to free ions, changes in these charges, obtained for each of the CIPs, are also included. All values are in e and are reported at the DFT level of theory

	***Q*(A)**								
			**CIPs**						
			**Na-Ade complexes**						
**Atom A**	**Free Ade⁻**		**N3N9**	**N9**	**N3**	**N7**	**N1**	**N10**	**π**
N3	-1.193		-0.024	0.001	-0.025	0.004	0.003	0.006	0.002
N1	-1.177		0.010	0.005	0.006	0.004	-0.014	0.002	0.002
N9	-1.166		-0.025	-0.023	-0.002	0.006	0.003	0.007	0.003
N7	-1.159		0.017	0.008	0.008	-0.019	0.002	0.006	0.003
N10	-1.105		-0.007	-0.004	-0.006	0.008	0.001	0.019	-0.004
C5	0.352		0.005	0.002	0.006	-0.003	0.004	0.006	-0.005
C4	0.838		-0.007	0.002	0.002	0.007	0.002	0.013	-0.010
C8	0.881		0.013	0.013	0.002	0.012	0.001	0.004	-0.001
C6	0.897		0.016	0.011	0.010	0.003	0.003	-0.039	0.006
C2	0.990		0.015	0.004	0.013	0.007	0.004	-0.001	0.000
H13	0.024		0.012	0.005	0.010	0.005	0.003	0.006	0.003
H14	0.024		0.012	0.009	0.005	0.009	0.002	0.005	0.003
H11	0.396		0.008	0.005	0.006	0.001	0.000	-0.003	0.003
H12	0.398		0.007	0.004	0.006	-0.004	0.002	-0.004	0.003
Q(Ade⁻)	–1.000	ΔQ(Ade⁻)	0.051	0.043	0.041	0.040	0.017	0.026	0.006
$Q(Na^+)$	1.000	$\Delta Q(Na^+)$	–0.052	–0.043	–0.041	–0.040	–0.017	–0.026	–0.006

Table S7 continues

Atom A			Q(A)						
			K-Ade complexes						
			N3N9			N7	N1	N10	
N3			-0.009	-	-	0.004	0.004	0.006	-
N1			0.007	-	-	0.003	-0.004	0.002	-
N9			-0.008	-	-	0.005	0.005	0.007	-
N7			0.011	-	-	-0.004	0.004	0.006	-
N10			-0.005	-	-	0.008	0.004	0.012	-
C5			0.004	-	-	-0.002	0.005	0.002	-
C4			-0.009	-	-	0.004	0.005	0.011	-
C8			0.009	-	-	0.006	0.002	0.004	-
C6			0.011	-	-	0.000	-0.004	-0.038	-
C2			0.011	-	-	0.004	-0.001	0.000	-
H13			0.008	-	-	0.004	0.003	0.006	-
H14			0.008	-	-	0.006	0.003	0.005	-
H11			0.006	-	-	0.000	0.000	0.001	-
H12			0.005	-	-	-0.004	0.003	0.000	-
$Q(Ade^{-})$		$\Delta Q(Ade^{-})$	0.050	-	-	0.036	0.030	0.025	-
$Q(K^{+})$	1.000	$\Delta Q(K^{+})$	–0.050			–0.036	–0.030	–0.024	

Explicit solvation model

The geometries for the out-of-plane N10-CIP is shown in Figure S1. The energy of the N10-CIP within the M-Ade-$(DMSO)_4$ systems was obtained by removing the explicit DMSO molecules and performing a single point energy calculation on the M-Ade complex. The energy of the N10-CIP is more than 5 kcal mol−1 less stable than the lowest energy N-CIP in the main text (Figure 2) and therefore won't be considered further in the main text.

Na-Ade-$(DMSO)_4$ molecular systems

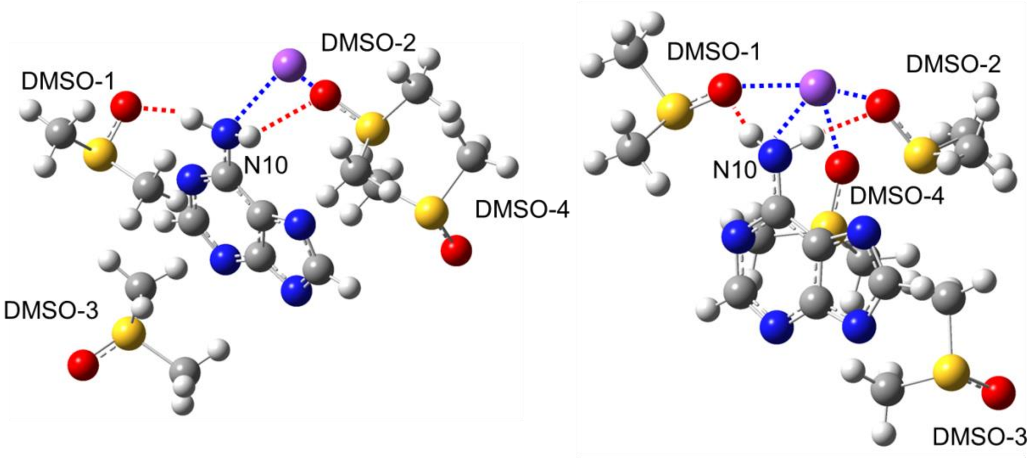

$\Delta E_{\text{Na-Ade}}$	N10, 5.8	N10-2, 7.3
E_f (ΔE_{system})	−37.0 (18.1)	−51.9 (3.2)

K-Ade-$(DMSO)_4$ molecular systems

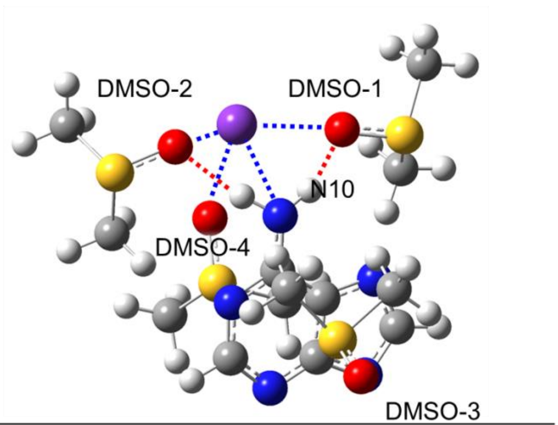

$\Delta E_{\text{Na-Ade}}$	N10, 13.7
E_f (ΔE_{system})	−51.1 (−1.5)

Figure S1: DFT-optimized structures of the indicated out-of-plane Na- and K-Ade-$(DMSO)_4$ systems and, relative to the lowest energy N-CIP in the main text (Figure 2), the energy difference $\Delta E_{\text{Na-Ade}}$ between Na-Ade complexes solvated by four DMSO molecules. The energy of formation (E_f) of the Na- and K-Ade-$(DMSO)_4$ molecular systems and, relative to the lowest energy system in the main text (Figure 2), the electronic energy difference between entire molecular systems (ΔE_{system}) are also provided. All values are in kcal mol^{-1}.

Interactions between DMSO solvent molecules and Na^+ and K^+ counter ions in M-Ade-$(DMSO)_4$ molecular systems

Table S8: The total interaction energy $E_{\mathrm{int}}^{\mathrm{M^+,DMSO}}$ and its covalent $V_{\mathrm{XC}}^{\mathrm{M^+,DMSO}}$ and electrostatic $V_{\mathrm{cl}}^{\mathrm{M^+,DMSO}}$ components computed for the for DMSO-1 and DMSO-2 solvent molecules and counter ions Na^+ and K^+ in the in-plane M-Ade-$(DMSO)_4$ molecular systems. All values in kcal mol^{-1} at the DFT level of theory.

	CIPs				
	**N3N9**	**N9**	**N3**	**N7**	**N1**
**Na-Ade-$(DMSO)_4$ molecular systems**					
$E_{\mathrm{int}}^{\mathrm{Na^+,DMSO\text{-}1}}$	2.3	2.5	3.4	-4.6	-44.4
$V_{\mathrm{XC}}^{\mathrm{Na^+,DMSO\text{-}1}}$	0.0	0.0	0.0	0.0	-9.9
$V_{\mathrm{cl}}^{\mathrm{Na^+,DMSO\text{-}1}}$	2.3	2.5	3.4	-4.6	-34.5
$E_{\mathrm{int}}^{\mathrm{Na^+,DMSO\text{-}2}}$	1.4	2.3	1.5	-38.4	-3.8
$V_{\mathrm{XC}}^{\mathrm{Na^+,DMSO\text{-}2}}$	0.0	0.0	0.0	-9.9	0.0
$V_{\mathrm{cl}}^{\mathrm{Na^+,DMSO\text{-}2}}$	1.4	2.3	1.5	-28.5	-3.8
**K-Ade-$(DMSO)_4$ molecular systems**					
$E_{\mathrm{int}}^{\mathrm{K^+,DMSO\text{-}1}}$	2.3			-2.6	-44.7
$V_{\mathrm{XC}}^{\mathrm{K^+,DMSO\text{-}1}}$	0.0			0.0	-13.1
$V_{\mathrm{cl}}^{\mathrm{K^+,DMSO\text{-}1}}$	2.3			-2.6	-31.6
$E_{\mathrm{int}}^{\mathrm{K^+,DMSO\text{-}2}}$	1.3			-34.1	-6.8
$V_{\mathrm{XC}}^{\mathrm{K^+,DMSO\text{-}2}}$	0.0			-11.7	0.0
$V_{\mathrm{cl}}^{\mathrm{K^+,DMSO\text{-}2}}$	1.3			-22.4	-6.8

Table S9: Intermolecular diatomic interaction energies between the M^+ counter ion (Na^+ and K^+) with the atoms A of the DMSO-3 solvent molecule of the in-plane M-Ade-$(DMSO)_4$ molecular systems at the DFT level of theory. All values in kcal mol^{-1}

Atom A of DMSO-3	CIPs				
	**N3N9**	**N9**	**N3**	**N7**	**N1**
	**Na-Ade-$(DMSO)_4$ molecular systems**				
S36	114.4	116.8	114.5	114.8	116.4
O37	-168.0	-172.2	-170.2	-172.8	-169.7
C38	-9.7	-10.5	-7.6	-10.6	-7.5
H39	4.3	4.7	3.8	6.2	4.0
H40	4.7	4.5	4.2	4.0	3.9
H41	5.7	7.4	4.1	7.3	4.0
C42	-7.5	-7.7	-9.5	-8.0	-10.3
H43	4.0	3.9	4.4	3.7	4.7
H44	4.0	3.9	4.3	3.4	4.8
H45	4.1	4.0	6.0	5.5	7.0
Total:	-43.9	-45.3	-46.0	-46.4	-42.9
	**K-Ade-$(DMSO)_4$ molecular systems**				
S36	104.4	-	-	104.5	106.5
O37	-148.9	-	-	-153.4	-154.3
C38	-7.2	-	-	-8.8	-9.3
H39	3.6	-	-	4.2	4.6
H40	3.5	-	-	3.9	4.3
H41	3.6	-	-	5.3	5.0
C42	-9.3	-	-	-7.3	-7.3
H43	4.2	-	-	3.6	3.6
H44	4.4	-	-	3.7	3.7
H45	5.7	-	-	3.7	3.7
Total:	-36.0	-	-	-40.5	-39.4

Table S10: Intermolecular diatomic interaction energies between the M^+ counter ion (Na^+ and K^+) with the atoms A of the DMSO-4 solvent molecule of the in-plane M-Ade-$(DMSO)_4$ molecular systems at the DFT level of theory. All values in kcal mol^{-1}

Atom A of DMSO-4	CIPs				
	**N3N9**	**N9**	**N3**	**N7**	**N1**
	**Na-Ade-(DMSO)₄ molecular systems**				
S36	116.0	114.6	116.5	70.5	113.5
O37	-169.5	-170.0	-172.8	-72.7	-166.2
C38	-7.6	-7.5	-7.7	-6.6	-7.7
H39	3.9	3.7	4.0	3.8	3.9
H40	3.9	4.0	3.9	2.7	4.1
H41	4.0	4.1	4.0	2.8	4.1
C42	-9.9	-9.9	-10.4	-11.4	-8.8
H43	4.6	4.4	4.5	4.0	4.2
H44	4.7	4.3	4.7	5.5	4.3
H45	6.5	5.8	7.4	7.4	5.2
Total:	-43.3	-46.6	-45.9	5.9	-43.2
	**K-Ade-(DMSO)₄ molecular systems**				
S36	102.5	-	-	47.3	61.8
O37	-149.5	-	-	-47.9	-60.6
C38	-8.8	-	-	-6.8	-10.3
H39	3.7	-	-	3.9	7.4
H40	4.1	-	-	2.8	3.7
H41	4.8	-	-	2.5	3.7
C42	-7.1	-	-	-5.4	-7.3
H43	3.6	-	-	2.1	2.7
H44	3.6	-	-	3.5	4.8
H45	3.7	-	-	2.3	2.8
Total:	-39.5	-	-	4.3	8.6

Interactions between DMSO solvent molecules and the adeninate anion Ade⁻ in M-Ade-(DMSO)₄ molecular systems

Table S11: The total interaction energy $E_{\mathrm{int}}^{\mathrm{Ade^-,DMSO}}$ and its covalent $V_{\mathrm{XC}}^{\mathrm{Ade^-,DMSO}}$ and electrostatic $V_{\mathrm{cl}}^{\mathrm{Ade^-,DMSO}}$ components computed for the for DMSO-3 and DMSO-4 solvent molecules and Ade⁻ in the in-plane M-Ade-(DMSO)₄ molecular systems. All values in kcal mol⁻¹ at the DFT level of theory.

	CIPs				
	**N3N9**	**N9**	**N3**	**N7**	**N1**
**Na-Ade-(DMSO)₄ molecular systems**					
$E_{\mathrm{int}}^{\mathrm{Ade^-,DMSO\text{-}3}}$	-13.5	-16.0	-15.9	-0.4	-19.7
$V_{\mathrm{XC}}^{\mathrm{Ade^-,DMSO\text{-}3}}$	-19.9	-17.4	-20.7	-8.9	-18.0
$V_{\mathrm{cl}}^{\mathrm{Ade^-,DMSO\text{-}3}}$	6.4	1.4	4.8	8.5	-1.7
$E_{\mathrm{int}}^{\mathrm{Ade^-,DMSO\text{-}4}}$	-13.3	-14.9	-15.2	-39.6	-20.4
$V_{\mathrm{XC}}^{\mathrm{Ade^-,DMSO\text{-}4}}$	-17.5	-21.1	-16.7	-27.4	-19.3
$V_{\mathrm{cl}}^{\mathrm{Ade^-,DMSO\text{-}4}}$	4.1	6.2	1.6	-12.2	-1.2
**K-Ade-(DMSO)₄ molecular systems**					
$E_{\mathrm{int}}^{\mathrm{Ade^-,DMSO\text{-}3}}$	-11.8			-25.2	-20.8
$V_{\mathrm{XC}}^{\mathrm{Ade^-,DMSO\text{-}3}}$	-15.5			-23.2	-17.6
$V_{\mathrm{cl}}^{\mathrm{Ade^-,DMSO\text{-}3}}$	3.8			-2.0	-3.2
$E_{\mathrm{int}}^{\mathrm{Ade^-,DMSO\text{-}4}}$	-14.0			-42.2	-25.0
$V_{\mathrm{XC}}^{\mathrm{Ade^-,DMSO\text{-}4}}$	-19.5			-29.8	-23.9
$V_{\mathrm{cl}}^{\mathrm{Ade^-,DMSO\text{-}4}}$	5.4			-12.5	-1.1
